# Interaction between androgen receptor and coregulator SLIRP is regulated by Ack1 tyrosine kinase and androgen

**DOI:** 10.1038/s41598-019-55057-2

**Published:** 2019-12-09

**Authors:** Dinuka De Silva, Zhentao Zhang, Yuanbo Liu, Joel S. Parker, Chenxi Xu, Ling Cai, Gang Greg Wang, H. Shelton Earp, Young E. Whang

**Affiliations:** 1 Department of Pathology and Laboratory Medicine, University of North Carolina at Chapel Hill, Chapel Hill, NC 27599 USA; 2 UNC Lineberger Comprehensive Cancer Center, University of North Carolina at Chapel Hill, Chapel Hill, NC 27599 USA; 3 Department of Hematology, Beijing Tiantan Hospital, Capital Medical University, Beijing, 100050 P.R. China; 4 Department of Genetics, University of North Carolina at Chapel Hill, Chapel Hill, NC 27599 USA; 5 Department of Biochemistry and Biophysics, University of North Carolina at Chapel Hill, Chapel Hill, NC 27599 USA; 6 Department of Medicine, University of North Carolina at Chapel Hill, Chapel Hill, NC 27599 USA; 7 Department of Pharmacology, University of North Carolina at Chapel Hill, Chapel Hill, NC 27599 USA; 8 Present Address: Urologic Oncology Branch, Center for Cancer Research, NCI, National Institutes of Health, Bethesda, MD 20892-1107 USA; 9 Present Address: Parkview Cancer Institute, 11050 Parkview Circle, Fort Wayne, IN 46845 USA

**Keywords:** Prostate cancer, Nuclear receptors

## Abstract

Aberrant activation of the androgen receptor (AR) may play a critical role in castration resistant prostate cancer. After ligand binding, AR is recruited to the androgen responsive element (ARE) sequences on the DNA where AR interaction with coactivators and corepressors modulates transcription. We demonstrated that phosphorylation of AR at Tyr-267 by Ack1/TNK2 tyrosine kinase results in nuclear translocation, DNA binding, and androgen-dependent gene transcription in a low androgen environment. In order to dissect downstream mechanisms, we searched for proteins whose interaction with AR was regulated by Ack1. SLIRP (SRA stem-loop interacting RNA binding protein) was identified as a candidate protein. Interaction between AR and SLIRP was disrupted by Ack1 kinase activity as well as androgen or heregulin treatment. The noncoding RNA, SRA, was required for AR-SLIRP interaction. SLIRP was bound to ARE’s of AR target genes in the absence of androgen. Treatment with androgen or heregulin led to dissociation of SLIRP from the ARE. Whole transcriptome analysis of SLIRP knockdown in androgen responsive LNCaP cells showed that SLIRP affects a significant subset of androgen-regulated genes. Our data suggest that Ack1 kinase and androgen regulate interaction between AR and SLIRP and that SLIRP functions as a coregulator of AR with properties of a corepressor in a context-dependent manner.

## Introduction

Prostate cancer remains the second leading cause of cancer death in men in the U.S. due to the development of resistance to androgen deprivation therapy. Prostate cancer cells remain dependent on the androgen receptor (AR) even in the castration resistant stage (with the rare exception of neuroendocrine tumor). The serum levels of the prostate specific antigen (PSA) protein increase in untreated castration resistant prostate cancer (CRPC) patients as reactivation of AR activity in prostate cancer cells drives the transcription of AR target genes such as PSA. Various mechanisms underlying AR reactivation in CRPC have been demonstrated^1,2^. They include AR gene overexpression/amplification, AR point mutations, AR splice variants and intratumoral androgen biosynthesis. Recently approved drugs such as abiraterone, enzalutamide, and apalutamide, potently inhibit AR signaling in CRPC tumor cells and led to improved outcome of CRPC patients^3^. However, CRPC inevitably develops resistance to these agents and therefore, metastatic prostate cancer continues to be a terminal disease.

The AR protein is a steroid receptor composed of three major domains, the N-terminal transactivation domain, the DNA-binding domain, and the C-terminal ligand binding domain^4^. Binding of ligands such as dihydrotestosterone (DHT) to AR causes nuclear translocation and binding to the androgen responsive elements (ARE) of the target genes and assembly of the active transcription complex that contains general transcription factors and AR coregulators^5^. These coactivators and corepressors modulate AR-induced gene expression in prostate cancer cells and may play a role in development and progression of prostate cancer. Coactivators recruit other proteins such as histone acetylases and other histone modifiers and chromatin remodeling complexes to promote transcription whereas corepressors are proposed to function by binding to unliganded receptor and recruiting histone deacetylases^6^. The nuclear receptor coactivator gene *NCOA2* (also known as *SRC2* or *TIF2*) is frequently amplified in prostate cancer tumors and overexpression of *NCOA2* may sensitize AR to be activated by low levels of androgen^7^. Conversely, expression of nuclear receptor corepressors *NCOR1* and *NCOR2* is decreased in metastatic prostate cancer, a finding highlighting the potential clinical relevance of androgen receptor corepressor/coactivator balance in prostate cancer^7,8^.

Another potential regulatory mechanism for AR activity is crosstalk with tyrosine kinase-dependent pathways. We have demonstrated that phosphorylation of AR at Tyr-267 by Ack1 (TNK2) nonreceptor tyrosine kinase results in nuclear translocation, DNA binding, and transactivation of target genes in the low androgen environment^9,10^. We hypothesized that Ack1 may affect the proteins interacting with AR and identified SLIRP as a candidate protein whose association with AR is regulated by Ack1. SLIRP (*S*RA stem-*l*oop *i*nteracting *R*NA binding *p*rotein) was initially isolated as a protein binding to the stem-loop structure of the RNA molecule SRA (steroid receptor RNA activator)^11^. SRA is postulated to act as a long noncoding RNA coactivator of nuclear receptors through its ability to form complexes with coactivators and corepressors^12,13^. SLIRP is a small protein (~13 kDa) composed mostly of the RNA recognition motifs and appears to function in nuclear receptor corepressor complexes^11^. In addition, SLIRP binds to LRPPRC protein in the mitochondria and acts as a global RNA chaperone involved in mitochondrial gene expression^14–16^. In this work, we show that SLIRP associating with AR is influenced by Ack1 kinase and androgen, which eject it from the AR complex, modulating AR signaling.

## Results

### AR interaction with SLIRP is disrupted by activated Ack1 kinase, androgen and heregulin treatment

**Figure 1:**
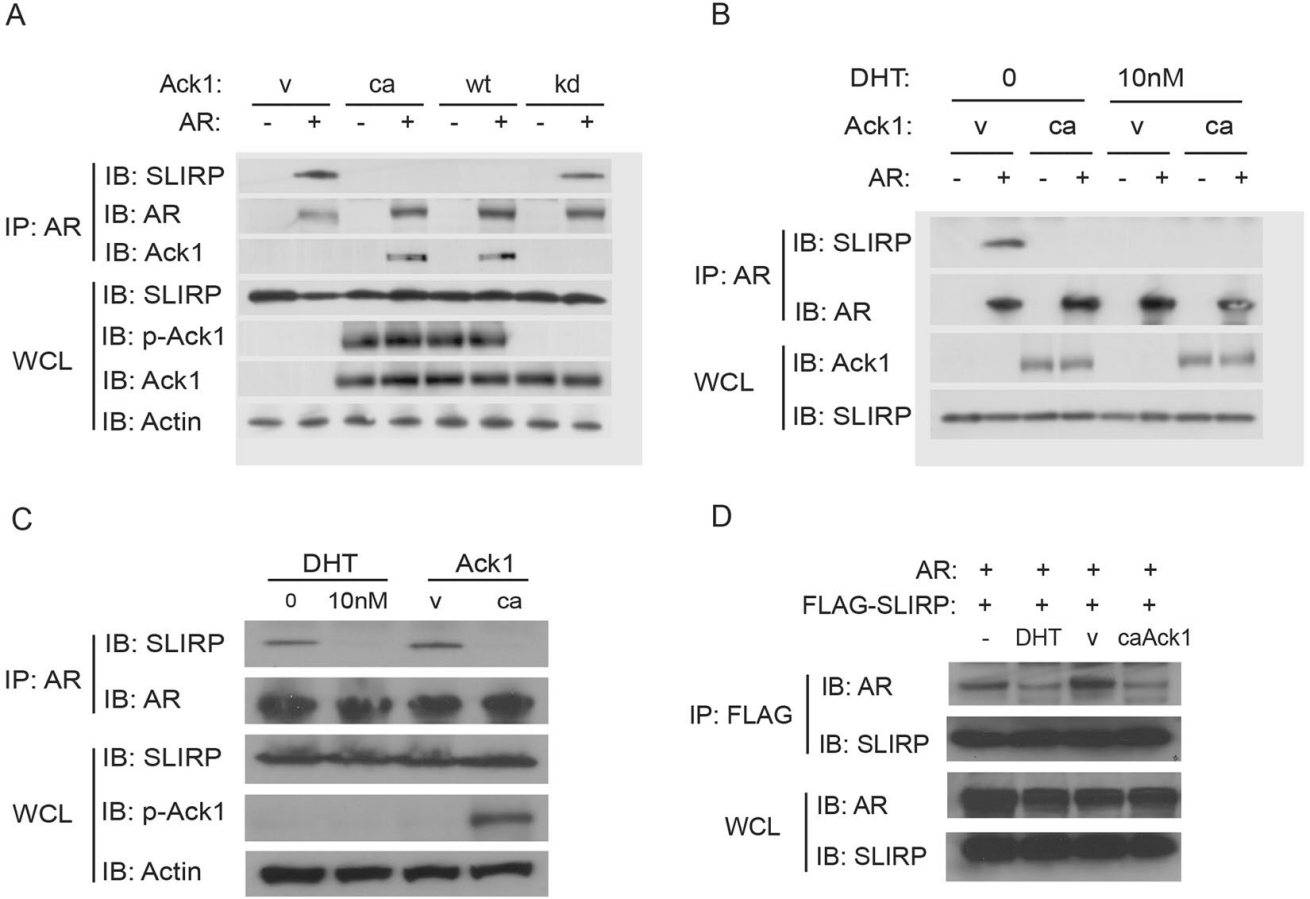
Ack1 and androgen ligand disrupt the interaction between AR and SLIRP. (**A**) Ack1 activation abrogates association between AR and SLIRP in 293 T cells. 293 T cells were transfected with the expression vectors encoding AR (1 μg), along with constitutively active Ack1 (ca) (1 μg), wild-type Ack1 (wt) (1 μg), kinase-dead Ack1 (kd) (1 μg), or empty vector (1 μg). After 24 hrs, protein extracts were immunoprecipitated with AR antibody and immunoblotted with antibodies against SLIRP, AR, or Ack1. Whole cell lysates (WCL) were immunoblotted with the indicated antibodies. (**B**) DHT treatment abrogates interaction of AR and SLIRP in 293 T cells. 293 T cells were transfected as indicated and treated with DHT (10 nM). After 24 hrs, immunoprecipitation and immunoblotting were performed using the indicated antibodies. (**C**) DHT treatment and Ack1 activation disrupt interaction between AR and SLIRP in LNCaP cells. For left two lanes, LNCaP cells were treated with DHT (10 nM) or vehicle for 24 hrs. For right two lanes, LNCaP cells were transfected with the expression vectors encoding constitutively active Ack1 (ca), or empty vector for 24 hrs. Protein extracts were immunoprecipitated with AR antibody and immunoblotted with antibodies against SLIRP or AR. Whole cell lysates were immunoblotted with the indicated antibodies. (**D**) Immunoprecipitation of SLIRP demonstrates that Ack1 and DHT disrupt interaction between AR and SLIRP. 293 T cells were transfected with the expression vectors encoding AR (1 μg) and FLAG-tagged SLIRP (1 μg), along with constitutively active Ack1 (1 μg) or empty vector (1 μg). Cells were treated with DHT (10 nM) or vehicle. After 24 hrs, protein extracts were immunoprecipitated with FLAG antibody and immunoblotted with the antibody against SLIRP. Whole cell lysates were immunoblotted with the indicated antibodies.

In an attempt to identify AR interacting proteins regulated by Ack1 kinase, we employed the differential in-gel electrophoresis (DIGE) approach to compare proteins co-immunoprecipitating with AR in 293 T cells transfected to express AR only versus AR and constitutively active Ack1 (Supplementary Fig. S1). SLIRP was identified as a candidate protein showing reduced association with AR in Ack1-expressing cells compared to cells expressing AR only. To confirm interaction between AR and SLIRP and the effect of Ack1 kinase activity, 293 T cells were transfected with the AR expression vector along with empty vector control, constitutively active Ack1 (caAck1), wild-type Ack1, or kinase-dead Ack1 (kdAck1). Endogenous SLIRP protein was co-immunoprecipitated with AR in vector control cells or kinase dead Ack1-expressing cells but not in cells with wild-type Ack1 overexpression or caAck1 overexpression (Fig. 1A). Treatment with DHT also led to loss of SLIRP co-immunoprecipitating with AR protein in 293 T cells (Fig. 1B). In androgen-sensitive LNCaP prostate cancer cells, immunoprecipitation of endogenous AR after DHT treatment or Ack1 expression demonstrated that association between AR and SLIRP is inhibited by DHT or Ack1 activation (Fig. 1C). In 293 T cells, FLAG-tagged SLIRP and AR were transfected and immunoprecipitation with FLAG antibody was performed. The amount of AR protein co-immunoprecipitating with SLIRP was decreased with DHT treatment or Ack1 expression (Fig. 1D). AR and SLIRP proteins were co-immunoprecipitated in castration-resistant C4-2 cells and DHT treatment decreased this interaction between AR and SLIRP in C4-2 cells (Supplementary Fig. S2). These data suggest that in the absence of androgen, there is stable association between AR and SLIRP, and that this interaction is disrupted by Ack1 kinase signaling or AR binding to androgen.

**Figure 2:**
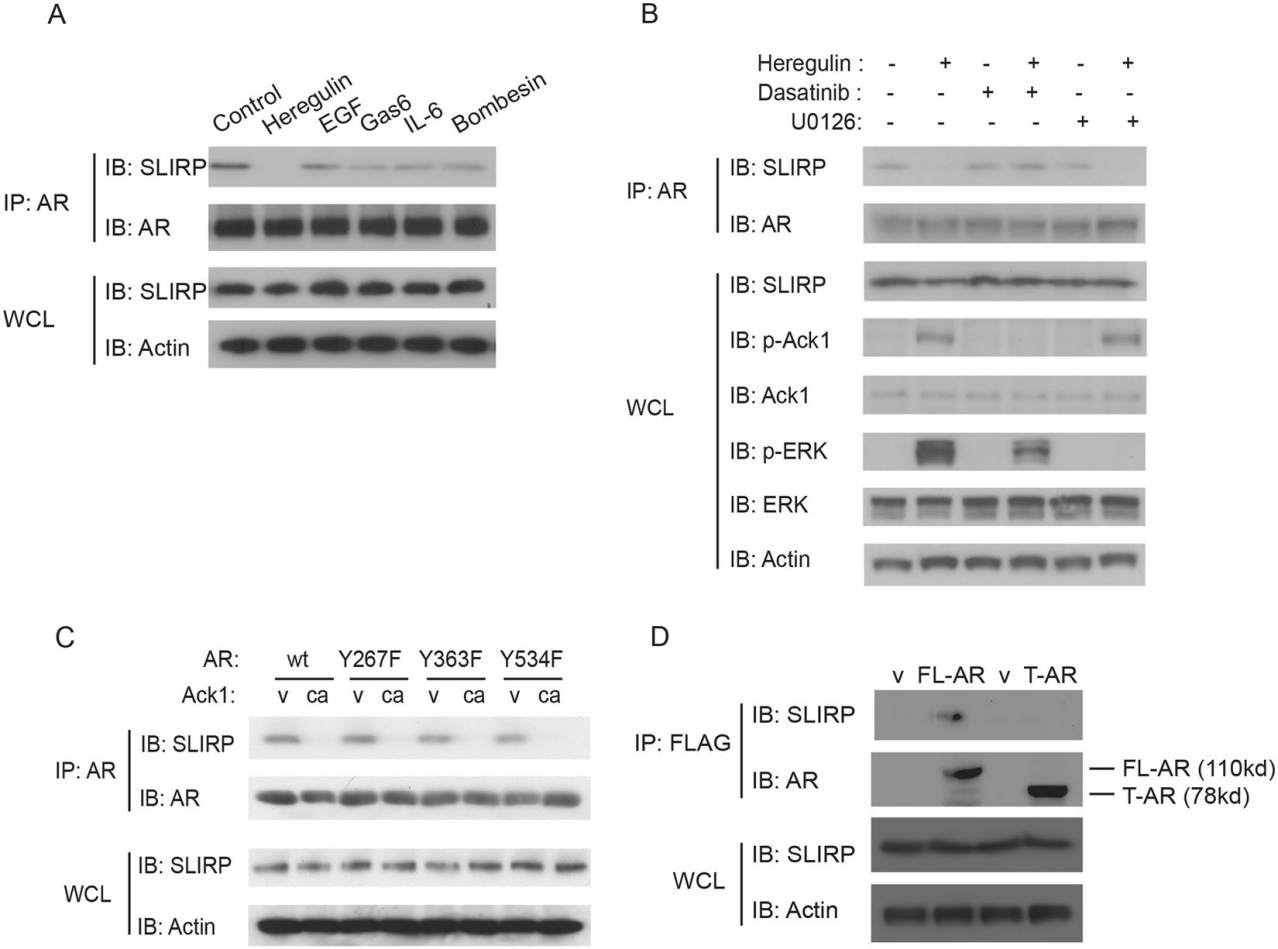
Heregulin treatment of LNCaP cells disrupts AR-SLIRP association through a non-Erk dependent pathway. (**A**) Heregulin induces loss of AR-SLIRP interaction. LNCaP cells were treated with heregulin (10 ng/ml), EGF (100 ng/ml), Gas6 (100 ng/ml), interleukin-6 (10 ng/ml) or bombesin (1 nM) for 60 min before harvesting. Protein extracts were immunoprecipitated with the AR antibody and immunoblotted with the indicated antibodies. Whole cell lysates were immunoblotted with the indicated antibodies. (**B**) Dasatinib but not U0126 inhibits heregulin-induced AR-SLIRP disassociation. LNCaP cells were pretreated with dasatinib (10 nM), U0126 (10 μM) or vehicles for 2 hrs before treatment with heregulin (10 ng/ml) for 60 min. Protein extracts were immunoprecipitated with the AR antibody and immunoblotted with the indicated antibodies. (**C**) Known AR tyrosine phosphorylation sites do not contribute to Ack1-induced AR-SLIRP dissociation. 293 T cells were transfected with the expression vectors encoding AR wild-type (wt), Y267F, Y363F, or Y534F (1 μg of each), along with constitutively active Ack1 (1 μg) or empty vector (1 μg). After 24 hrs, protein extracts were immunoprecipitated with the AR antibody and immunoblotted with the antibodies against SLIRP or AR. (**D**) Lack of the ligand-binding domain of AR impairs AR-SLIRP interaction. 293 T cells were transfected with the expression vectors encoding FLAG-tagged full-length (FL) AR (2 μg), truncated (T) AR (amino acids 1–660) (2 μg), or empty vector (2 μg). After 24 hrs, protein extracts were immunoprecipitated with the FLAG antibody and immunoblotted with the antibodies against SLIRP or AR. Immunoblotting of whole cell extracts was performed as indicated.

We have previously shown that treatment of prostate cancer cells with heregulin induces downstream activation of Ack1 kinase and AR phosphorylation at Tyr-267 while treatment with epidermal growth factor, interleukin-6 and bombesin (gastrin-releasing peptide) induces Src kinase activation and AR phosphorylation at Tyr-534^9^. Treatment of LNCaP cells with heregulin led to loss of interaction between SLIRP and AR, but treatment with epidermal growth factor, Gas6, interleukin-6 or bombesin had little or no effect on SLIRP-AR association (Fig. 2A). Treatment with dasatinib (which inhibits Ack1 kinase) inhibited heregulin-induced disruption of SLIRP-AR association (Fig. 2B). However, treatment with MEK inhibitor U0126 that inhibits Erk activation did not affect heregulin-induced disruption of SLIRP-AR association. This result is consistent with the notion that heregulin-induced disruption of SLIRP-AR association may involve HER-2 and downstream Ack1 but not the MEK-Erk pathway downstream. Since SLIRP does not contain any tyrosine residues, we tested the effect of mutating known AR tyrosine phosphorylation sites (Fig. 2C). Loss of AR tyrosine phosphorylation sites had no effect on the regulation of AR-SLIRP association, as AR Tyr-267, Tyr-363, and Tyr-534 mutants exhibited loss of association with SLIRP, similarly to wild-type AR, when constitutively active Ack1 was expressed. Thus, the specific component controlled by Ack1 activity is unknown, but since the loss of SLIRP from the complex is not seen with kinase dead Ack1, tyrosine phosphorylation of an unidentified protein may be required. The truncated AR mutant (amino acid 1–660) lacking the ligand-binding domain did not associate with SLIRP (Fig. 2D).

### Noncoding RNA SRA is essential in AR-SLIRP interaction

**Figure 3:**
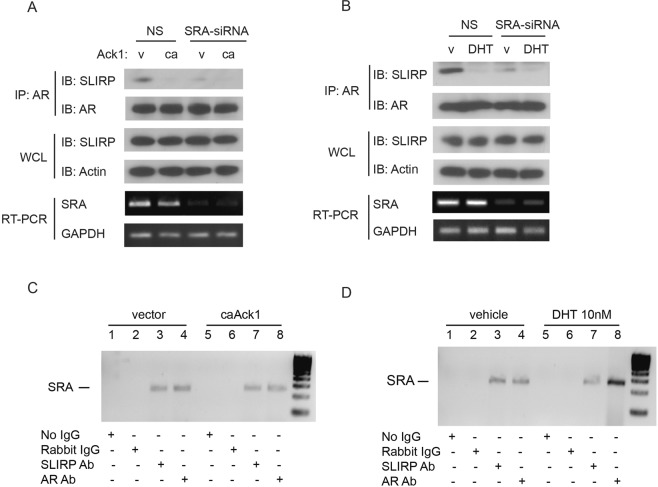
Noncoding RNA SRA is essential for AR-SLIRP interaction. (**A**) SRA knockdown abrogates the interaction between AR and SLIRP. LNCaP cells were transfected with SRA-siRNA or nonsense RNA (50 nM) and expression vectors encoding constitutively active Ack1 (ca), or empty vector (2 μg). After 48 hrs, protein extracts were immunoprecipitated with the AR antibody and immunoblotted with the antibody against SLIRP or AR. Whole cell lysates were immunoblotted with the indicated antibodies. SRA and glyceraldehyde 3-phosphate dehydrogenase (GAPDH) mRNA levels were determined by RT-PCR. (**B**) LNCaP cells were transfected with SRA-siRNA or nonsense RNA (50 nM) and were treated with DHT (10 nM) or vehicle. After 48 hrs, protein extracts were immunoprecipitated with the AR antibody and immunoblotted with the antibodies against SLIRP or AR. Whole cell lysates were immunoblotted with the indicated antibodies. SRA and GAPDH mRNA levels were determined by RT-PCR. (**C**) Ack1 activation does not affect the association between SRA-SLIRP and SRA-AR. LNCaP cells were transfected with expression vectors encoding constitutively active Ack1 (ca), or empty vector (2 μg) for 24 hrs. Protein extracts were immunoprecipitated with no IgG, control rabbit IgG, antibody against SLIRP or AR. RNA was isolated from the precipitated fraction and the SRA RNA level was detected by RT-PCR. (**D**) Androgen increases the association between SRA-AR, but not SRA-SLIRP. LNCaP cells were treated with DHT (10 nM) for 12 hrs. Immunoprecipitation and RT-PCR for SRA were perfomed as described above.

It has been postulated that the noncoding RNA coactivator SRA, acting as a scaffold, mediates association between SLIRP and nuclear receptors^11^. Therefore, we investigated the role of SRA in AR-SLIRP interaction by using the siRNA specific for SRA (Supplementary Fig. S3A). SRA knockdown abrogated the interaction between AR and SLIRP in LNCaP cells as effectively as Ack1 or DHT (Fig. 3A and B). SRA RNA levels were moderately increased by DHT or Ack1 (Supplementary Fig. S3B), and this finding rules out the decrease or loss of SRA as a potential mechanism for inhibition of AR-SLIRP interaction by Ack1 or DHT. SRA RNA was detectable after immunoprecipitation of AR and SLIRP and this is consistent with the model of SRA being in the same complex containing both AR and SLIRP. Ack1 did not affect the amount of SRA RNA associating with AR or SLIRP proteins (Fig. 3C). DHT treatment increased the SRA associating with AR but not with SLIRP (Fig. 3D).

### SLIRP represses AR signaling

**Figure 4:**
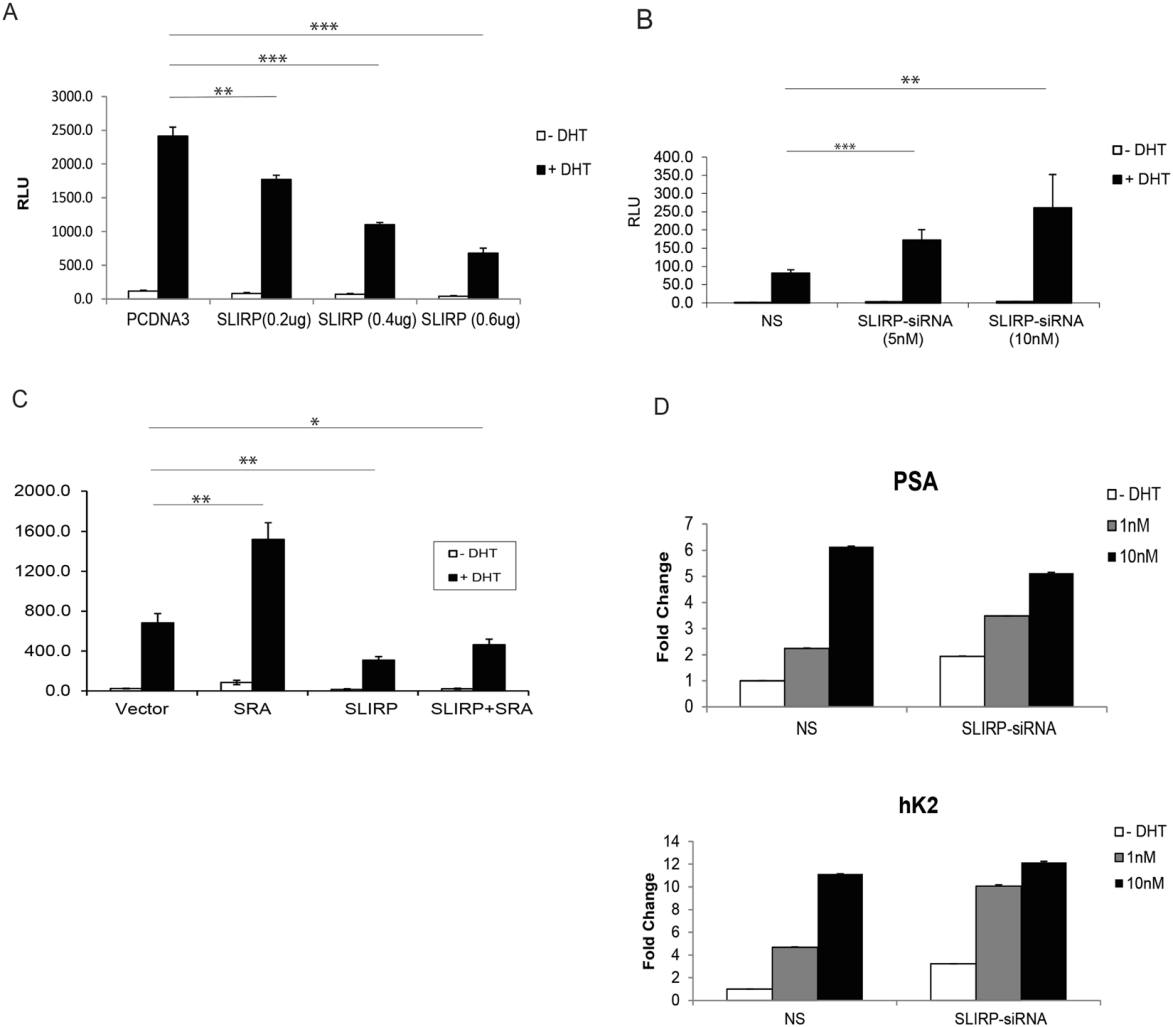
SLIRP represses AR transcriptional activity. (**A**) SLIRP expression inhibits AR reporter activity. LNCaP cells were transfected with AR (0.1 μg), ARR2PB-Luciferase (0.1 μg), and SLIRP (0.2 μg, 0.4 μg, and 0.6 μg) for 24 hrs and then treated with 1 nM DHT or vehicle control for another 24 h. Values present the mean ± SEM (n = 3). (**B**) SLIRP knockdown increases AR transcriptional activity in a dose dependent manner. LNCaP cells were co-transfected with AR(0.1 μg), ARR2PB-Luciferase (0.1 μg), and SLIRP-siRNA (5 nM, 10 nM) for 24 hrs and treated with 1 nM DHT or vehicle control for another 24 hrs. Values present the mean ± SEM (n = 3). (**C**) Effect of SRA on AR transactivity. LNCaP cells were co-transfected with AR(0.1 μg), ARR2PB-Luciferase(0.1 μg), along with either SRA expressing vector (0.3 μg), SLIRP (0.3 μg) or SRA plus SLIRP for 24 hrs. Cells were then treated with 1 nM DHT or vehicle control for another 24 hrs. Values present the mean ± SEM (n = 3). (**D**). SLIRP knockdown increases AR mediated transcriptional activity of PSA and hK2. Real-time PCR of genes PSA and hK2 with SLIRP knockdown (40 nM) and 1 nM DHT treatment. Values present the mean ± SEM (n = 3).

**Figure 5:**
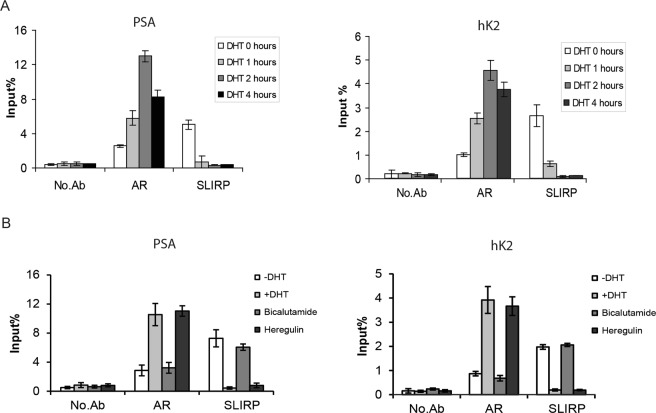
Effects of DHT and Ack1 activation on SLIRP recruitment to the androgen response element (ARE). (**A**) DHT treatment impairs the recruitment of SLIRP to the androgen responsive enhancers. LNCaP cells were treated with DHT (10 nM) for the indicated time. Chromatin immunoprecipitation analysis for binding of AR and SLIRP proteins to the ARE III enhancer of PSA and hK2 genes was performed. The amount of precipitated DNA was determined by quantitative PCR. (**B**) Heregulin but not bicalutamide impairs the recruitment of SLIRP to the androgen responsive enhancers. LNCaP cells were treated with vehicle control, DHT (10 nM), bicalutamide (5 μM), or heregulin (10 ng/ml) for 2 hrs. Chromatin immunoprecipitation analysis for AR and SLIRP binding to PSA and hK2 enhancer DNA was performed.

We investigated the functional significance of SLIRP expression in the AR signaling pathway by characterizing the effect of SLIRP on AR reporter activity. SLIRP expression led to dose-dependent reduction of AR reporter activation induced by androgen (Fig. 4A). Conversely, SLIRP knockdown resulted in increased AR reporter activity (Fig. 4B). SRA expression activated AR reporter, as expected from its role as a coactivator. When SRA and SLIRP are both expressed, SLIRP repressed SRA-induced AR activation (Fig. 4C). To confirm the role of SLIRP as a repressor, we determined the effect of SLIRP knockdown on endogenous AR target gene expression. SLIRP knockdown resulted in increased mRNA levels of canonical AR target genes PSA or human kallikrein 2 (hK2, also known as *KLK2*) in the absence or suboptimal dose of androgen, but less at the full dose (Fig. 4D,E). We next characterized the recruitment of SLIRP to upstream androgen responsive enhancers (ARE) of these two genes. Chromatin immunoprecipitation analysis in LNCaP cells demonstrated that androgen treatment led to recruitment of AR to the ARE regions of PSA and hK2 (Fig. 5A,B). In the absence of DHT, SLIRP was bound to the ARE, but DHT treatment (but not bicalutamide, an AR antagonist) led to loss of SLIRP binding to the ARE (Fig. 5C,D). Heregulin treatment led to AR recruitment to the ARE and loss of SLIRP binding to the ARE (Fig. 5). This result is consistent with our previous finding that heregulin treatment activates HER-2 and downstream Ack1 activation^10^ and suggests that either Ack1 or DHT causes dissociation of AR and SLIRP. In the absence of androgen, there is a basal amount of the complex of AR and SLIRP bound to the ARE of target genes in LNCaP cells. Androgen or Ack1 kinase enhances AR binding to the ARE while dissociating SLIRP from this molecular complex. Chromatin immunoprecipitation analysis performed in castration-resistant C4-2 cells shows that in contrast to androgen-sensitive LNCaP cells, AR protein is constitutively bound to the ARE in the absence of androgen, but SLIRP binding to the ARE is reduced by androgen (Supplementary Fig. S4).

### Whole transcriptome analysis demonstrates that SLIRP affects a significant subset of androgen-regulated genes

**Figure 6:**
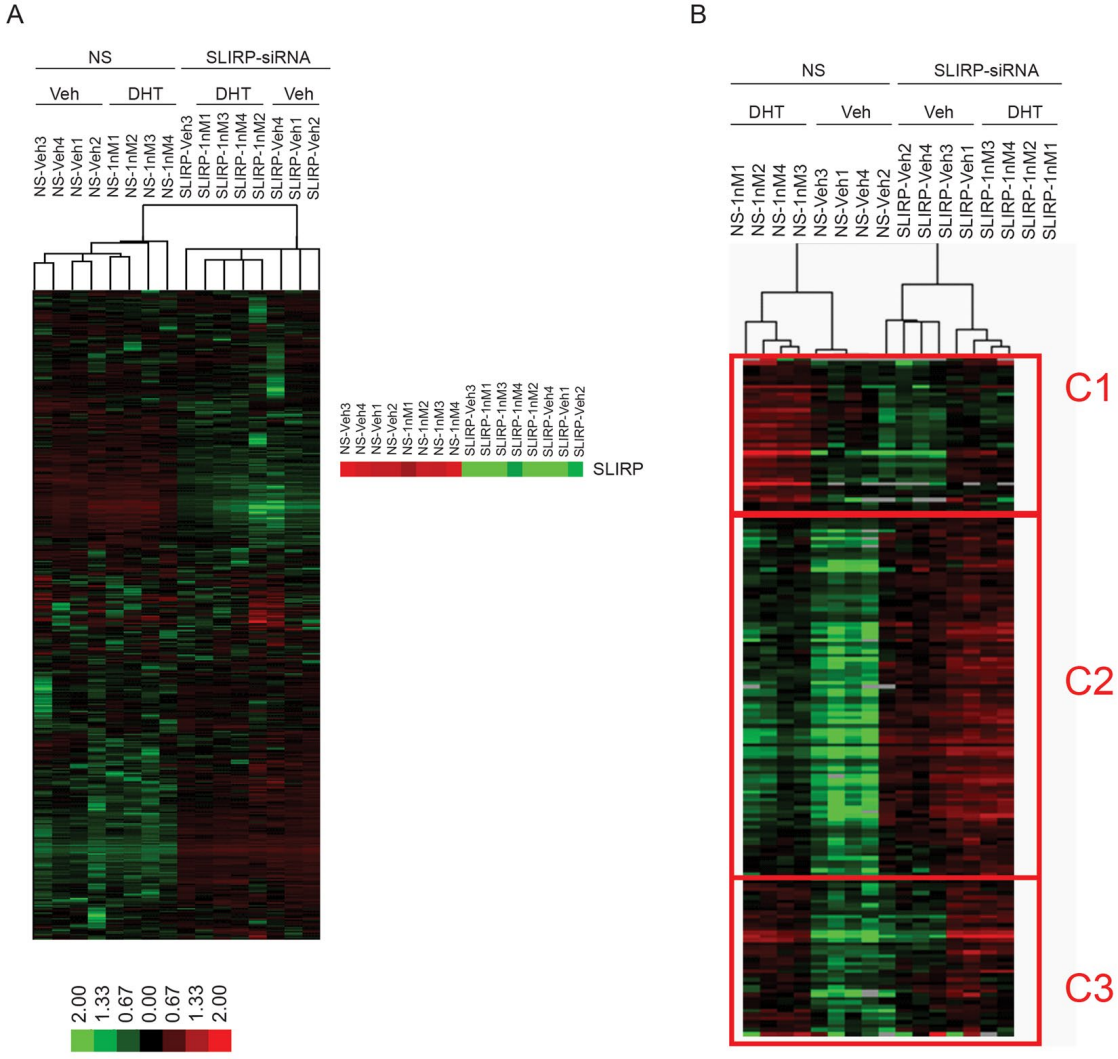
Whole transcriptome analysis identifies three groups of AR target genes regulated by SLIRP. LNCaP cells were transfected with nonsense control siRNA or SLIRP-siRNA and then treated with vehicle or 1 nm DHT for another 24 hrs. RNA isolated from 4 replicates of each treatment condition and subjected to high throughput sequencing. (**A**) The heat map of unsupervised hierarchical cluster analysis is shown. The highlighted expression level of SLIRP show the effect of SLIRP siRNA. (**B**) Clustering analysis using the signature of 176 androgen-regulated genes identified three classes of gene expression patterns that respond differentially to loss of SLIRP. Loss of SLIRP increased androgen-induced expression in C2 genes whereas loss of SLIRP led to decreased androgen-induced expression in C1 genes. Loss of SLIRP had little or no effect on C3 genes.

We performed whole transcriptome analysis by RNA sequencing of LNCaP cells in which SLIRP was knocked down with siRNA. Since the impact of SLIRP knockdown was more pronounced at the suboptimal androgen concentration (Fig. 4D,E), we chose 1 nM DHT for AR stimulation. When nonsense control (vehicle and 1 nM DHT) was compared to SLIRP knockdown (vehicle and 1 nM DHT), 2253 genes were upregulated and 1563 genes were downregulated by SLIRP knockdown (Fig. 6A and Supplementary Fig. S5A). The altered genes from the RNA-seq data were analyzed by **D**atabase for **A**nnotation, **V**isualization and **I**ntegrated **D**iscovery (DAVID) and Ingenuity Pathway analysis (IPA) software to explore biological diseases and pathways that were significantly affected by the loss of SLIRP. DAVID highlighted significantly enriched gene ontology like acetylation which plays a role in epigenetic regulation (Supplementary Fig. S5B). Top biological functions from IPA include cell survival and RNA post transcriptional modification. Both DAVID and IPA analysis identified cell cycle as an important biological function affected by SLIRP (Supplementary Fig. S5C).

Comparison between vehicle control and androgen treatment (1 nM DHT) in cells transfected with nonsense RNA generated a list of 176 androgen-regulated genes under suboptimal stimulation (Supplementary Table). Hierarchical analysis of this 176 androgen-regulated gene signature applied to the RNA-seq data of all four groups revealed three classes of genes that respond differentially to loss of SLIRP (Fig. 6B). The expression pattern of gene classes C1 and C2 is affected by loss of SLIRP. In the C2 class (composed of 92 genes out of 176 genes, representing 52% of genes) (Supplementary Table), loss of SLIRP led to increased expression levels under both the basal condition and the androgen-stimulated conditions. This result is consistent with the idea that SLIRP acts predominantly as a corepressor of AR for androgen-induced genes. In the other class (C1, 43 genes or 24% of genes) (Supplementary Table), loss of SLIRP led to reduced androgen-induced gene expression. For C1 genes, SLIRP appears to be required for androgen-stimulated gene expression and might be postulated to act as a coactivator of AR. In the third class (C3, 41 genes or 23%) (Supplementary Table), loss of SLIRP had no or little effect on androgen-induced gene expression (Supplementary Table). For C3 genes, SLIRP may not play a role in androgen-regulated gene expression. Taken together, these data show that SLIRP affects multiple biological processes in addition to AR and that SLIRP regulates expression of a significant subset of androgen-regulated genes.

## Discussion

In this report, we show that complex formation between SLIRP and AR can be regulated by androgen or by activated Ack1 kinase and heregulin treatment. In the absence of ligand- or kinase-induced activation, there is a stable complex including SLIRP and AR and presumably the RNA component SRA. Androgen stimulation, Ack1 kinase, and heregulin treatment (presumably by activating the ErbB2 receptor and Ack1 downstream) lead to dissociation of SLIRP from AR. In the absence of androgen, SLIRP is constitutively bound to the enhancer elements of the canonical androgen-regulated genes (e.g. PSA and hK2). After androgen stimulation or treatment with heregulin (but not bicalutamide), AR is recruited to the enhancer while SLIRP dissociates from the enhancer. Other well-characterized AR corepressors NCOR and SMRT display similar behavior as they are recruited to the DNA by bicalutamide but not by androgen^17,18^. The finding that association between AR and SLIRP is disrupted by Ack1 is similar to the effect of Src on another AR corepressor LCoR (ligand-dependent corepressor)^19^. Interaction of LCoR and AR has been shown to be inhibited by Src tyrosine kinase, which activates AR, in part, through phosphorylation of AR at Tyr-534^9,20,21^. Therefore, SLIRP displays properties of a bona fide corepressor of AR. However, similar to many nuclear receptor coregulators, SLIRP affects a broad array of nuclear receptors, including estrogen receptor^11^. Binding of AR to corepressors NCOR and SMRT involves direct interaction between AR and the corepressor^22–24^. In this work, we demonstrated that association between AR and SLIRP requires the SRA RNA molecule. Lanz *et al*. reported that SRA is found in the ribonucleoprotein complex containing nuclear receptor and that SRA can be co-immunoprecipitated by the AR antibody^12^. Therefore, we hypothesize that AR-SLIRP interaction may be indirect and the SRA molecule may be a necessary component for bringing AR and SLIRP together into the same complex. Mechanisms by which Ack1 or heregulin leads to dissociation between SLIRP and AR are unclear. It is unlikely to involve direct tyrosine phosphorylation of AR or SLIRP. SLIRP protein does not contain tyrosine residues. AR mutants in which Tyr-267, −363- and −534 have been mutated behave in the same way as wild-type AR. It is postulated that Ack1 kinase-induced or ligand-induced change in the AR conformation leads to assembly of the active AR transcription complex and “exclusion” of SLIRP from this complex. It’s possible that SLIRP may bind (directly or indirectly) to the same site that Ack1 uses to bind to AR. Also, the SLIRP-SRA complex associating with AR may represent a small fraction of the SRA bound to AR. But detailed mechanisms underlying this process remain to be characterized. AR splice variant 7 missing the ligand-bind domain has emerged as a mediator of resistance to androgen signaling inhibitors abiraterone and enzalutamide^25^. Our data show the lack of interaction between SLIRP and the truncated AR 1–660 mutant (structurally similar to AR-V7). This may provide an additional explanation for the constitutively activated nature of AR-V7^26,27^.

The role of SLIRP as an AR corepressor is also supported by whole transcriptome analysis showing that expression of the majority (92 out of 176 or 52%) of androgen-regulated genes are increased by loss of SLIRP. The fact that loss of SLIRP increases expression of these genes in the absence of androgen stimulation raises a possibility that loss of SLIRP facilitates nuclear localization and activity of AR. Our data showing that loss of SLIRP had no effect on some AR-responsive genes and led to decreased expression of other androgen-regulated genes are consistent with a recent report on a comprehensive analysis of androgen receptor coregulators in prostate cancer^28^. Liu *et al*.^28^ characterized the effect of knocking down 18 clinically relevant AR coregulators on the expression of the 452 AR target gene signature in LNCaP cells. Knockdown of p300 had the broadest impact on gene expression, affecting 57% of AR target genes. Knockdown of NCOA1 and NCOA2 affected only 11% and 10% of genes. Interestingly, knockdown of coregulators simultaneously increased expression of certain genes while decreasing expression of other genes. Our data on SLIRP, in agreement with Liu *et al*.^28^, suggest that each AR coregulator affects only a subset of AR target genes. However, since the two studies used different conditions for androgen induction (e.g. 1 nM DHT in our study vs 5 nM R1881 in Liu *et al*.^28^), the specific numbers and proportions of genes may not be comparable. In addition to its role in regulating AR and other nuclear receptors, SLIRP is likely to be involved in several other processes such as mitochondrial gene expression^15,16^. We found that SLIRP knockdown affects expression of more than 3000 genes. SLIRP knockout mice are viable, but have minimally reduced fertility^29^.

The role of SLIRP in prostate cancer progression was investigated by exploratory analysis of clinical specimens. Query of the cBioPortal for Cancer Genomics (http://cbioportal.org)^30^ for alteration of SLIRP in prostate cancer revealed no point mutation of SLIRP. Analysis of currently available data in cBioPortal showed that 6.9% of primary tumors (84 out of 1121) demonstrated *SLIRP* gene copy number loss while 11.4% of metastatic tumors (105 out of 918) demonstrated *SLIRP* gene copy number loss (Supplementary Information Table 1). The difference is statistically significant (p = 0.003 by Chi-square), and this result is consistent with the hypothesis that *SLIRP* loss promotes prostate cancer progression. However, some studies in cBioPortal also report *SLIRP* gene amplification (22 out of 1052 tumors or 2% in 3 largest studies^31–33^). The role of SLIRP in clinical progression of prostate cancer is uncertain and will require more investigation.

In summary, SLIRP has been identified as an AR-associated protein and the interaction between AR and SLIRP is disrupted by Ack1 kinase and androgen and heregulin treatment. Loss of SLIRP increases the expression of the majority of androgen-induced genes although expression of some genes is reduced by loss of SLIRP. The precise role of SLIRP in prostate cancer remains to be elucidated.

## Materials and Methods

### Cells and reagents

LNCaP cells and 293 T cells were obtained from the American Type Culture Collection (Manassas, VA, USA). EGF (R&D Systems, Minneapolis, MN, USA), IL-6 (R&D Systems), Gas6 (R&D Systems) and bombesin (Sigma-Aldrich, St Louis, MO, USA), U0126 (Cell signaling, Beverly, MA, USA) were purchased. Heregulin was a gift from Genentech (South San Francisco, CA, USA). Dasatinib was a gift from Bristol-Myers-Squibb (Princeton, NJ, USA). A mouse monoclonal antibody against AR (F39.4.1, Biogenex, San Ramon, CA, USA) was used for immunoblotting and a polyclonal antibody against AR (C-19, Santa Cruz) was used for immunoprecipitation. The antibody against total Ack1 was described previously^34^. A phospho-specific antibody against Ack1 p-Tyr-284 (#09–142) was obtained from Millipore (Billerica, MA, USA). Antibody against SLIRP (#ab51523) was purchased from Abcam (Cambridge, MA, USA). Antibodies against total ERK (#9102) and phospho-ERK (#9101) were obtained from Cell Signaling Technology (Beverly, MA, USA). Actin antibody (#A3853) and anti-Flag affinity gel (#A2220) were purchased from Sigma-Aldrich (St. Louis, MO, USA).

### Plasmids

The plasmids encoding AR, wild-type (wt) Ack1, kinase dead (kd) Ack1, constitutively active (ca) Ack1, ARR2-PB-luciferase reporter were previously described^34^. Flag-SLIRP and SRA expressing vector were purchased from Origene Inc. (Rockville, MD, USA). Y267F, Y363F, Y534F mutants of AR were constructed using Stratagene QuikChange™ Site-Directed Mutagenesis Kit (La Jolla, CA, USA), as previously described^35^.

### Immunoprecipitation, immunoblotting, and chromatin Immunoprecipitation (ChIP)

Cells were lysed in lysis buffer containing 50 mmol/L Tris-HCl, 0.1% NP40, 150 mmol/L NaCl, 10% glycerol, 2 mmol/L EDTA, plus proteinase inhibitor (Roche Diagnostic, Indianapolis, IN, USA) and phosphatase inhibitor (St. Louis, MO, USA). Immunoprecipitation was done by incubating the mixture of 500 μg protein lysis with 2 μg IgG and 50 μL protein A agarose beads (santa cruz biotechnology, Santa Cruz, CA, USA) overnight at 4 °C. Immunoprecipitated fraction was resolved on 4–12% Bis-tris gel (Invitrogen, Carlsbad, CA, USA). For immunoblottting, antibodies were prepared at 1:1000 dilution unless specifically indicated. ChIP analysis was performed following the protocol described before^10^. Briefly, an antibody against AR (Santa Cruz Technology, Santa Cruz, CA) or SLIRP (Abcam, Cambridge, MA, USA) was applied to immunoprecipitate DNA which is associated with AR and SLIRP. DNA was subjected to quantitative PCR using the primers and the probe targeting the distal ARE III enhancer sequence of the PSA gene or the primers and the probe targeting the distal enhancer of the hK2 gene as described previously^10^.

### Transfections and knockdown

Both LNCaP cells and 293 T cells were transfected with expression vectors encoding AR, Ack1, or Flag-SLIRP using Effectene (Qiagen, Valencia, CA, USA) according to the manufacturer’s instructions. To knock down SLIRP and SRA, validated Stealth RNAi siRNA against SLIRP and SRA (Invitrogen, Carlsbad, CA, USA) was used according to the manufacturer’s instructions. 24 h after transfection, cells were treated with ligands in serum-free media as indicated.

### Differential in gel electrophoresis – mass spectrum (DIGE-MS) assay

293 T cells in 10 cm culture dish were transfected with the expression vectors encoding AR (1 μg), or AR (1 μg) plus constitutively active Ack1 (1 μg) using Effectene. After 24 h, protein extracts were harvested and immunoprecipiated with AR antibody as described above. The precipitant was analyzed using one-dimensional DIGE method in UNC Systems-Proteomics Core Facility, following protocols described previously^36^. The spot of interested was picked and analyzed by mass spectrometry at the UNC Proteomics Core Facility. The resulting peptides were mixed with matrix (α-Cyano-4-Hydroxycinnamic Acid) and analyzed using a MALDI-TOF/TOF mass spectrometer (Applied Biosystems 4800 Plus). MS spectra were obtained in reflector positive ion mode and peaks with signal-to-noise ratio above 20 were selected for MS/MS analysis (maximum of 45 MS/MS spectra per spot). All spectra were searched using GPS Explorer Software Version 3.6 (Applied Biosystems) linked to the Mascot (Matrix Science, Inc.) search engine.

### Reporter gene assays and quantitative reverse-transcription PCR

LNCaP cells (8 × 10^4^ cells per well/12well plate) were transfected with the ARR2-PB-luciferase reporter (0.1 μg) along with the AR expression vector (0.1 μg), and vector (3 μg) or SLIRP (3 μg) using Effectene, as described previously^9^. After overnight incubation, cells were pretreated with 1 nM DHT as indicated for 24 h. Luciferase activity was determined as described previously. For measurement of PSA and hK2 mRNA levels, LNCaP cells were transfected with 40 nM siRNA using Effectene for 24 h. Next day, the cells were treated with 1 nM DHT in serum-free medium for 24 h. Total RNA was isolated and the mRNA levels of PSA and hK2 were determined by quantitative reverse-transcription PCR, as described previously^10^. To detect the level of SRA RNA, total RNA was isolated using TRI Reagent (Molecular Research Center, Inc, Cincinnati, OH, USA) per manufacturer’s instruction. First-strand cDNA was synthesized using oligo-dT primers. Quantitative PCR analysis was performed in triplicate using Cybergreen master mix (Roche Diagnostic, Indianapolis, IN, USA) in the ABI PRISM 7900HT system (Applied Biosystems, Foster City, CA). The primer sequence were obtained from Agoulnik *et al*.: forward, 5′TCTACTGGTGCAAGAGCTTTCAAG3′; reverse, 5′ATGAGGGAGCGGTGGATGT3′^37^.

### Immunoprecipitation RT-PCR Assay

This assay was done following the protocol described elsewhere^11^. Briefly, cell lysate from treated LNCaP cells was immunoprecipitated with SLIRP IgG, AR IgG, non-specific Rabbit IgG, or no IgG. Total RNA was isolated from immunoprecipitated fraction using TRI Reagent and SRA RT-PCR was performed as described above.

### Whole transcriptome analysis

LNCaP cells were transfected with custom nonsense control siRNA (sense-GUUCAGGUCGAUAUGUGCA) or a pool of SLIRP siRNA (HSS130109, HSS188666, HSS188667 Invitrogen, Carlsbad, CA) at 40 nM for 24 hrs. Cells were washed once with phosphate-buffered saline and replaced with serum-free medium containing EtOH or 1 nM DHT for another 24 hrs. RNA was collected using RNeasy kit (Qiagen, Valencia, CA). RNA concentration was checked by Nanodrop and quality assayed by Bioanalyzer. Two biological replicates were collected from 2 different experiments for a total of 4 replicates. RNA collected was sequenced using Illumia HiSeq. 2000 single read 50 base pair by the UNC High Throughput Sequencing Facility (reference genome hg19) and aligned and normalized by the Bioinformatics core. Resulting dataset was analyzed by SAM and the up/down-regulated gene list generated with an FDR ≤ 1 were used in DAVID functional software^38,39^ and Ingenuity to generate pathway maps (IPA, QIAGEN Redwood City, www.qiagen.com/ingenuity). Genelists were also generated for AR signature using SAM by comparing NS-vehicle versus NS-1nM DHT. Heatmap was generated by using Cluster (Stanford University) and Java Treeview (Sourceforge). Sequencing data are available in the Gene Expression Omnibus database under the accession number GSE77829.

### Statistical analysis

Transfection and luciferase data are shown as mean ± SEM. Statistical analysis was performed using Student’s *t* test, with a *p* value of <0.05 regarded as significant.

## Supplementary information

Supplementary Table

Supplementary Information

## Data Availability

The datasets generated and analysed during the current study are available in the Gene Expression Omnibus repository, accession number GSE77829.
