# Supplementary material for: Interaction between androgen receptor and coregulator SLIRP is regulated by Ack1 tyrosine kinase and androgen
Source: Sci Rep. 2019 Dec 9;9:18637. doi: 10.1038/s41598-019-55057-2 (PMC6901447; doi:10.1038/s41598-019-55057-2)
Supplement: Supplementary file 2 — Supplementary Information [file 41598_2019_55057_MOESM2_ESM.pdf]

Interaction between androgen receptor and coregulator SLIRP is regulated by Ack1 tyrosine kinase and androgen

Dinuka De Silva, Zhentao Zhang, Yuanbo Liu, Joel S. Parker, Chenxi Xu, Ling Cai,

Gang Greg Wang, H. Shelton Earp, and Young E. Whang

Supplementary Figure Legends

Supplementary Fig. S1: Differential in gel electrophoresis analysis in 293T cells. 293T cells were transfected with the expression vectors encoding AR (1µg), or AR (1µg) plus constitutively active Ack1 (1µg). After 24 hrs, protein extracts were harvested and immunoprecipitated with AR antibody. The precipitant was analyzed using DIGE method [1]. Briefly, the proteins precipitated from cells transfected with the AR expression vector were labeled with the Cy3 dye while the proteins precipitated from cells transfected with the AR vector plus the Ack1 vector were labeled with the Cy5 dye. Equal amounts of Cy3- and Cy5-labeled proteins were also combined into one sample. The three samples (Cy3-labeled proteins, Cy5-labeled proteins, and the combined "merged" sample) were run on gel electrophoresis. The highlighted band showing the highest level of differential expression was cut out and processed for mass spectroscopy. Several different proteins were identified and SLIRP was selected for further characterization.

Supplementary Fig. S2: Interaction between AR and SLIRP in castration resistant C4-2 cells. A) Protein extracts from C4-2 cells were immunoprecipitated with control IgG antibody or anti-AR antibody, then immunoblotted with antibodies against SLIRP or AR. Unprecipitated whole cell lysates (WCL) were immunoblotted with antibodies against SLIRP or AR. B) C4-2 cells were treated with DHT (10nM) or vehicle for 16 hrs. Protein extracts were immunoprecipitated with control IgG antibody or anti-AR antibody, then immunoblotted with antibodies against SLIRP or AR, as indicated. Whole cell lysates were immunoblotted with antibodies against SLIRP or AR.

Supplementary Fig. S3: (A) SRA knockdown by SRA-siRNA. 293T cells were transfected with various doses of SRA-siRNA (25nM, 50nM, 100nM) and non-sense (NS) control. mRNA was isolated and quantitative RT-PCR was done to analyze SRA RNA levels. Values present the means ± SEM (n=3). (B) SRA

expression with DHT treatment or Ack1 activation. LNCaP cells were transfected with 2µg of expression vectors encoding constitutively active Ack1 (ca), empty vector, and with DHT (10nM) and vehicle control for 24 hrs, respectively. mRNA was isolated and real-time RT-PCR was done to analyze SRA RNA levels. Values present the mean ± SEM (n=3).

Supplementary Fig. S4: Effect of DHT on SLIRP recruitment to the androgen response element (ARE) in castration resistant C4-2 cells. C4-2 cells cultured in charcoal-stripped medium were treated with DHT (10nM) for 2 hrs. Chromatin immunoprecipitation analysis for binding of AR (panel A) and SLIRP (panel B) proteins to the ARE enhancers of PSA and hK2 genes was performed. The amount of precipitated DNA was determined by quantitative PCR.

Supplementary Fig. S5: LNCaP cells were transfected with nonsense control siRNA or SLIRP-siRNA for 24 hrs and then treated with vehicle or 1nm DHT (n=4) for another 24 hrs. RNA was isolated and subjected to high throughput RNA sequencing. A) When NS-Veh and NS-1nM DHT (group 1) were compared to SLIRP-Veh and SLIRP-DHT samples (group 2) using SAM software, 2253 genes were upregulated and 1563 genes were down-regulated with an FDR ≤ 1%. B) Functional pathways of the gene list generated using DAVID annotation database. C) Ingenuity (IPA) analysis of gene list to explore the biological significance of SLIRP loss. Pathways such as cell cycle and cell survival are the most affected by SLIRP loss.

REFERENCE

1 Alzate, O. *et al.* Proteomic identification of brainstem cytosolic proteins in a neuropathic pain model. *Brain Res Mol Brain Res* **128**, 193-200, doi:10.1016/j.molbrainres.2004.06.037 (2004).

Supplemental Figure 1

A

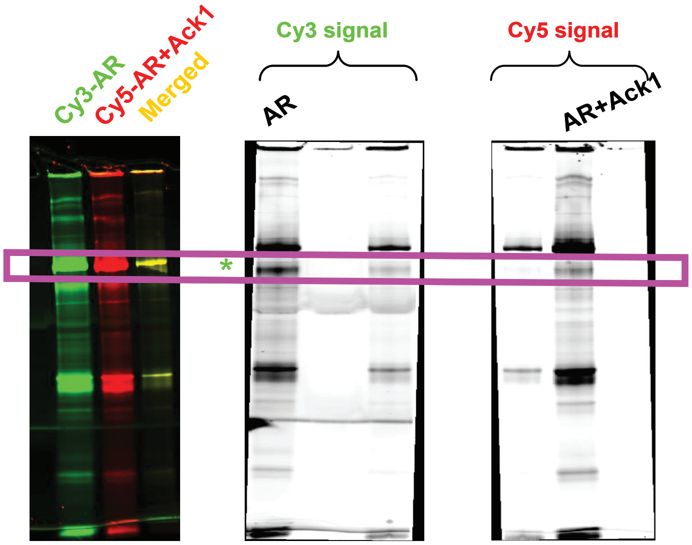

Supplemental Figure 2

A

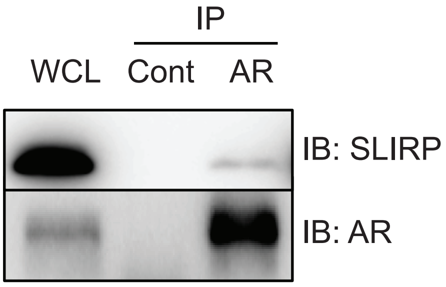

B

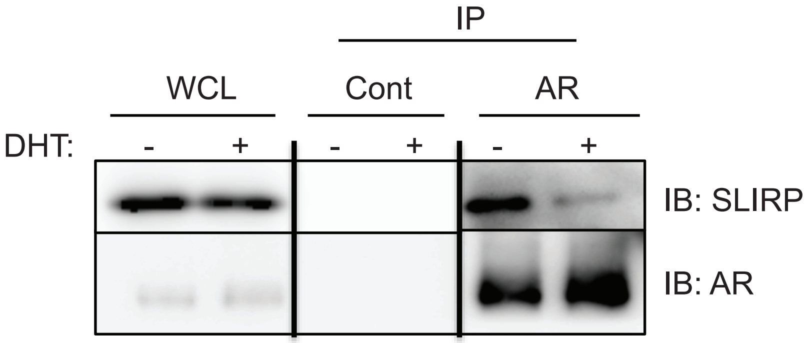

Supplemental Figure 3

A

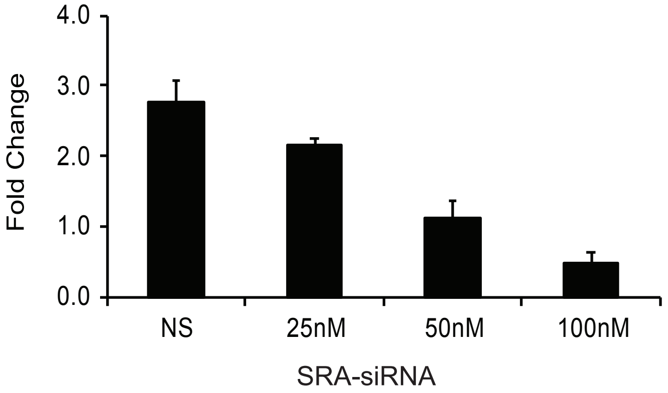

B

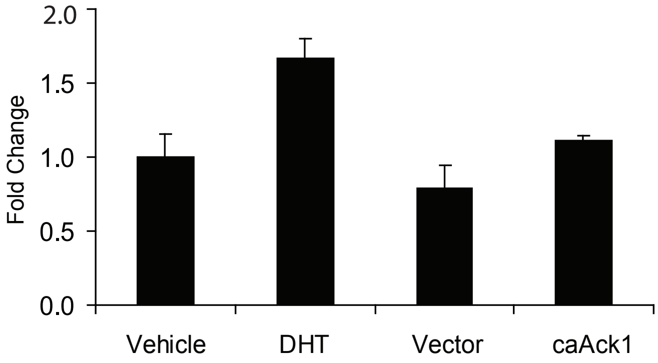

Supplemental Figure 4

A

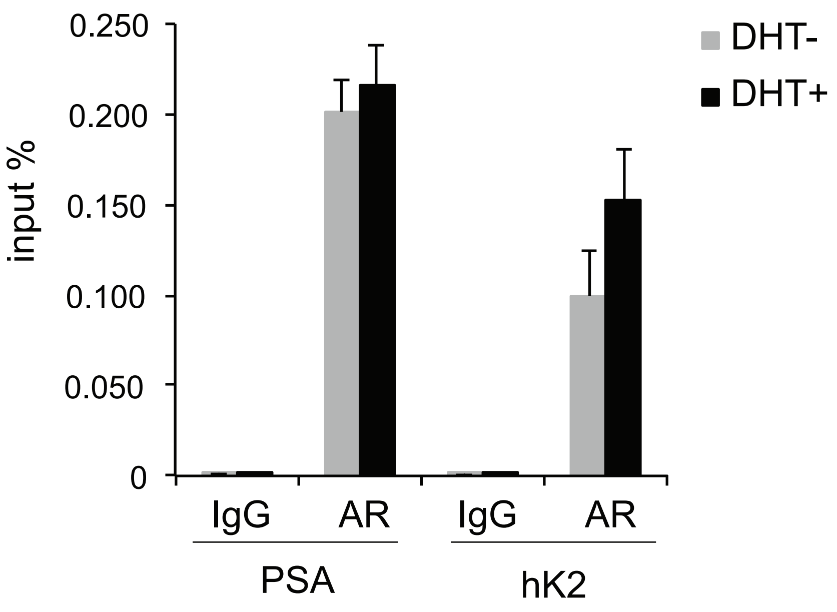

B

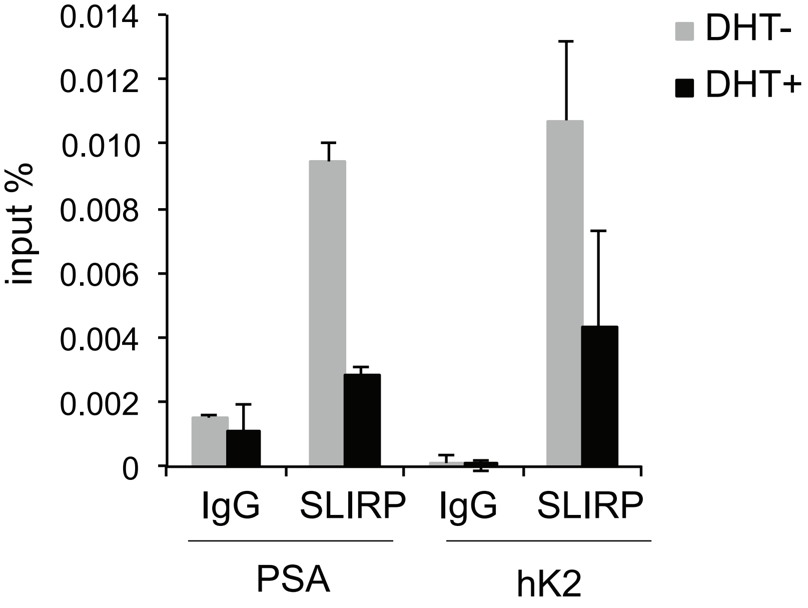

Supplemental Figure 5

A

Up Regulated genes	Down Regulated genes
2253	1563

FDR ≤ 1%

B

Up - Regulated genes

Category	Term	RT	Genes	Count	%	P - Value	Benjamini
SP_PIR_KEY WORDS	acetylation	RT		754	3.1	5.5E -157	3.4E -154
SP_PIR_KEY WORDS	phosphoprotein	RT		1387	5.7	2.0E -148	6.2E -146
SP_PIR_KEY WORDS	nucleus	RT		924	3.8	2.5E -111	5.1E -109
SP_PIR_KE YWORDS	cell cycle	RT		184	0.8	3.5E -57	5.4E -55

Down - Regulated genes

Category	Term	RT	Genes	Count	%	P - Value	Benjamini
SP_PIR_KEY WORDS	phosphoprotein	RT		738	4.4	3.1E -19	1.9E -16
SP_PIR_KEY WORDS	endoplasmic reticulum	RT		127	0.8	2.7E -18	8.4E -16
GOTERM_BP _FAT	protein localization	RT		142	0.8	6.6E -15	7.5E -12
GOTERM_BP _FAT	protein transport	RT		127	0.8	1.7E -14	1.4E -11

C

**Diseases and Disorders**	
Name	***p - Value***
Cancer	3.09E -25 - 2.32E -04
Infectious disease	3.26E -22 - 4.64E -05
Gastrointestinal disease	2.40E -17 - 1.73E -04

**Molecular and Cellular Functions**	
Name	***p-Value***
Cell Cycle	1.15E-28-2.39E-04
Cell Death and Survival	8.55E-25-2.07E-04
Cellular assembly and organization	3.99E-24-1.54E-04
DNA Replication, Recombination, Repair	3.99E-24-2.41E-04
RNA post-trancriptional modification	1.54E-22-2.41E-04

Study ID	Primary	Metastasis
prad_mich[2]	1/11	2/50
prad_su2c_2019[3]	NA	38/444
prad_su2c_2015[4]	NA	17/118
nepc_wcm_2016[5]	NA	7/107
prad_broad_2013[6]	3/55	0/1
prad_broad[7]	1/109	NA
prad_fhcrc[8]	5/18	38/115
prad_mskcc[9]	1/72	3/9
prad_mskcc_2014[10]	3/104	NA
prad_p1000[11]	Data Unavailable	Data Unavailable
prad_eururol_2017[12]	0/64	NA
prad_tcga_pub[13]	29/290	NA
prad_tcga_pan_can_atlas_2018[14]	41/488	NA
prad_mskcc_2017[15]	Data Unavailable	Data Unavailable
prad_mpcproject_2018[16]	0/5	0/14

Supplementary Table 1: Specimens with *SLIRP* gene loss in primary and metastatic tumors
The association between *SLIRP* loss and tumor malignancy was explored using cBioPortal[1] platform. Using the cBioPortal's annotated copy-number data, either deep deletion (a homozygous deletion) or shallow deletion (a heterozygous deletion) of *SLIRP* gene was used to categorize subjects in 'SLRIP loss' group.

References

1. Gao, J. *et al.* Integrative Analysis of Complex Cancer Genomics and Clinical Profiles Using the cBioPortal. *Sci. Signal.* **6**, pl1–pl1 (2013).
2. Grasso, C. S. *et al.* The mutational landscape of lethal castration-resistant prostate cancer. *Nature* **487**, 239–243 (2012).
3. Abida, W. *et al.* Genomic correlates of clinical outcome in advanced prostate cancer. *Proc. Natl. Acad. Sci.* **116**, 11428–11436 (2019).
4. Robinson, D. *et al.* Integrative clinical genomics of advanced prostate cancer. *Cell* **161**, 1215–1228 (2015).
5. Beltran, H. *et al.* Divergent clonal evolution of castration-resistant neuroendocrine prostate cancer. *Nat. Med.* **22**, 298–305 (2016).
6. Baca, S. C. *et al.* Punctuated evolution of prostate cancer genomes. *Cell* **153**, 666–677 (2013).
7. Barbieri, C. E. *et al.* Exome sequencing identifies recurrent SPOP, FOXA1 and MED12 mutations in prostate cancer. *Nat. Genet.* **44**, 685–689 (2012).
8. Kumar, A. *et al.* Substantial interindividual and limited intraindividual genomic diversity among tumors from men with metastatic prostate cancer. *Nat. Med.* **22**, 369–378 (2016).

9. Taylor, B. S. *et al.* Integrative genomic profiling of human prostate cancer. *Cancer Cell* **18**, 11–22 (2010).
10. Hieronymus, H. *et al.* Copy number alteration burden predicts prostate cancer relapse. *Proc. Natl. Acad. Sci. U. S. A.* **111**, 11139–11144 (2014).
11. Armenia, J. *et al.* The long tail of oncogenic drivers in prostate cancer. *Nat. Genet.* **50**, 645–651 (2018).
12. Ren, S. *et al.* Whole-genome and Transcriptome Sequencing of Prostate Cancer Identify New Genetic Alterations Driving Disease Progression. *Eur. Urol.* (2017). doi:10.1016/j.eururo.2017.08.027
13. Cancer Genome Atlas Research Network. The Molecular Taxonomy of Primary Prostate Cancer. *Cell* **163**, 1011–1025 (2015).
14. Hoadley, K. A. *et al.* Cell-of-Origin Patterns Dominate the Molecular Classification of 10,000 Tumors from 33 Types of Cancer. *Cell* **173**, 291-304.e6 (2018).
15. Abida, W. *et al.* Prospective Genomic Profiling of Prostate Cancer Across Disease States Reveals Germline and Somatic Alterations That May Affect Clinical Decision Making. *JCO Precis. Oncol.* **2017**, (2017).
16. Metastatic Prostate Cancer Project. Available at: https://mpcproject.org/data-release. (Accessed: 29th July 2019)
